# Birds combined calls more than 11 million years ago

**DOI:** 10.1038/s41598-025-05497-w

**Published:** 2025-07-01

**Authors:** Ambre Salis, Robin J. Ryder, Axel Molina, Philippe Schlenker, Emmanuel Chemla

**Affiliations:** 1 Département d’Etudes Cognitives, Ecole Normale Supérieure, Institut Jean-Nicod (ENS - EHESS - CNRS), PSL University, Paris, France; 2 Department of Life Sciences, Imperial College London, Buckhurst Road, Ascot, SL57PY, UK; 3 Department of Mathematics, Imperial College London, London, UK; 4 Department of Linguistics, New York University, New York, USA; 5 Département d’Etudes Cognitives, Ecole Normale Supérieure, LSCP (ENS - EHESS - CNRS), PSL University, Paris, France; 6 Earth Species Project, Berkeley, California USA

**Keywords:** Animal communication, Combination, Evolution, Mobbing, Syntax, Ancestral signals, Behavioural ecology, Cultural evolution, Animal behaviour

## Abstract

Multiple members of the tit and chickadee (= Parid) family combine two classes of calls, F and D, in a rigid order FD. In Japanese tits, FD has been argued on the basis of multiple experiments to involve syntax and non-trivial compositionality. How ancient are these call combinations? We show that FD combinations (as well as individual F and D calls) are present in nearly all Parid species, and almost absent in their closest relatives, the Remizidae and Stenostiridae. Using phylogenetic tools and ancestral reconstruction methods, we infer that FD combinations very likely emerged between 11 and 26 million years ago in the eastern Himalayas. This result contributes to evolutionary animal linguistics using a comparative phylogenetic approach to reconstruct the evolution of call combinations.

## Introductions

Our understanding of the communication systems of *modern* animal species has become increasingly precise thanks to the development of ambitious formal analyses and of correspondingly sophisticated experimental tests. At the same time, the emergence of large, participatory databases (e.g., xeno-canto, Macaulay library) has given researchers access to a large quantity of recordings, even if these are less carefully curated than is feasible for field studies by a single research group. Together, precise fieldwork and large-scale data collection offer excellent terrain for comparative research. Phylogenetic relations among species are also, in many cases, well established thanks to DNA studies, and modern technologies and statistics thus allow the reconstruction of ancient systems and of their evolutionary dynamics. In this area, recent studies have traced back the ‘boom’ calls of some monkeys (the cercopithecines) more than 5 million years ago^1^, the snake-related calls of two bird species approximately 8 million years ago^2^, the gagaga calls of herring gulls^3^ approximately 5.5. million years ago, and they have described the evolutionary path of the drum pecking behavior in woodpeckers starting ~ 22.5 million years ago^4^.

While research on the phylogenetic reconstruction of elementary calls is underway, the evolution of call combinations remains largely unexplored. Call combinations are increasingly described in modern species of mammals^5,6^ and birds^7^, but also in insects^8^. These combinations are beginning to be explored from a semantic and syntactic point of view. For example, among African monkeys, putty-nosed monkeys *Cercopithecus nictitans* have been argued to have call sequences that are combinatorial but not compositional [^9^, but see ^10^ for alternative analyses], while two Campbell’s monkey *Cercopithecus campbelli* calls have been argued to have a root-suffix structure, possibly with a compositional semantics^10,11^. Research on the meaning of these combinations has been very enlightening, suggesting in some cases that the meaning of these combinations was not just the conjunction of the meanings of their parts^12^. Our knowledge of the evolution of these combinations remains, however, limited. Recent work on birds has explored the evolution of their songs (e.g., by confirming the presence of female songs in the common ancestor of songbirds^13^) characterized by complex combinations of notes. But songs, unlike calls, are thought not to involve individually meaningful components. Understanding how combinations of specific calls arise, with each possessing a distinct meaning, represents a form of evolutionary animal linguistics, a territory that remains currently underexplored^14^.

Of particular interest for the present study are bird call combinations. For example, Japanese tit (*Parus minor)* mobbing calls combine high frequency notes (hereafter designed as ‘F’ notes) with broadband, noisy notes (hereafter designed as ‘D’ notes). In isolation, F notes have been suggested to be alert calls, while D notes appear to be recruitment calls^15^. A series of experiments suggests that this combination of calls is an instance of non-trivial compositionality, in the sense that the meaning of FD is derived from the meaning of F and D (hence the combination is compositional), but without being reducible to the mere conjunction of these two calls (hence the compositionality is non-trivial) [^16,17^, see ^18^ for a complete review].

Call combinations are not specific to the Japanese tit, as a large number of Parids (tits and chickadees) combine high frequency notes with noisy, broadband elements to form similar FD calls (the term ‘chickadee’ originating from the combination of ‘chick’ notes (= F) and ‘dee’ elements (= D)^19^). The term ‘F’ note is here used as an umbrella term that may include several types of notes, such as the notes typically referred to as A, B, and C in North-American chickadees^20^. Some researchers may use an even broader definition of what may count as F notes, and accordingly as FD combinations^19^, which we will not do here to keep our later analysis on the conservative side of the field. Details of production and perception of this FD combination appear to depend on the species. For example, the Carolina chickadee *Poecile carolinensis* produces a chick-a-dee call used in a large variety of contexts. It is the proportion of each note that seems to inform receivers about different situations (e.g., foraging, anti-predator, flock cohesion^20,21^). Delving into the semantics of this combination presents a promising avenue for further research; however, there is also a need for comprehensive comparative studies on the production of this combination across the entire clade. Understanding the evolutionary history of such a call is crucial for exploring the semantic variation within each species, and posit hypotheses on the mechanisms responsible for this variation. In addition, call order, and specifically the FD call, has recently been explored as a mechanism facilitating heterospecific communication^22^. By testing how pervasive this combination is in Parids, we can gain crucial information on this signal mechanism. Older comparative works have described the presence or absence of the FD combination in most Parid species^19^; but their conclusions were limited by the statistical and methodological constraints of their time. In this article, we present the first phylogenetic analysis of a call combination in birds.

## Results

We collected a total of 6h17min of recordings, from 52 species of Paridae, Remizidae and Stenostiridae (the two closest clades to the Paridae). Each recording (519 files) contained between 2 and 148 calls (M = 16.50, SD = 14.93), for a total of 8,562 calls, accounting for 1h17min of the recordings. A majority of these calls contained a single note (*n* = 2,550, 29.8%) or the same note repeated (*n* = 2,210, 25.8%). The rest of the calls (*n* = 3,802, 44.4%) were combinations of at least two distinguishable notes. See Materials and Methods for details.

**Fig. 1:**
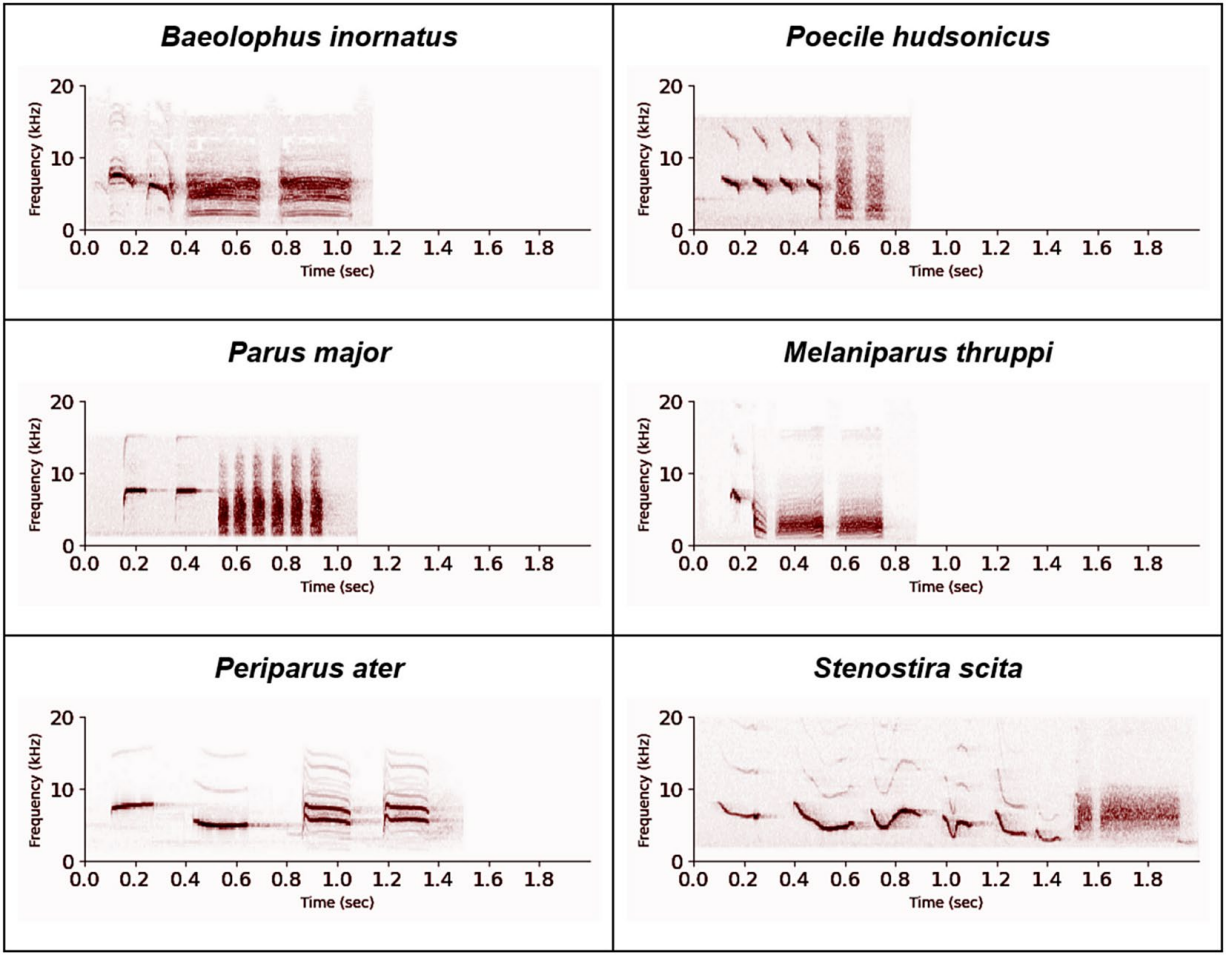
Examples of FD calls from six different species. First, four species for which FD calls were detected in large numbers: the oak titmouse (*Baeolophus inornatus*), the boreal chickadee (*Poecile hudsonicus*), the great tit (*Parus major*), the acacia tit (*Melaniparus thruppi*). We also add the coal tit (*Periparus ater*, a species with relatively few FD calls in the Paridae) and the fairy flycatcher (*Stenostira scita*, the one species with FD calls outside of the Paridae). Spectrograms were obtained using the stft (Short-Time Fourier Transform) function from scipy.signal in python, using a window size of 256 samples (nperseg), an overlap of 128 samples (noverlap), and an FFT length of 256 points. The display uses a power scaling factor of 0.6 for best contrast.

**Fig. 2:**
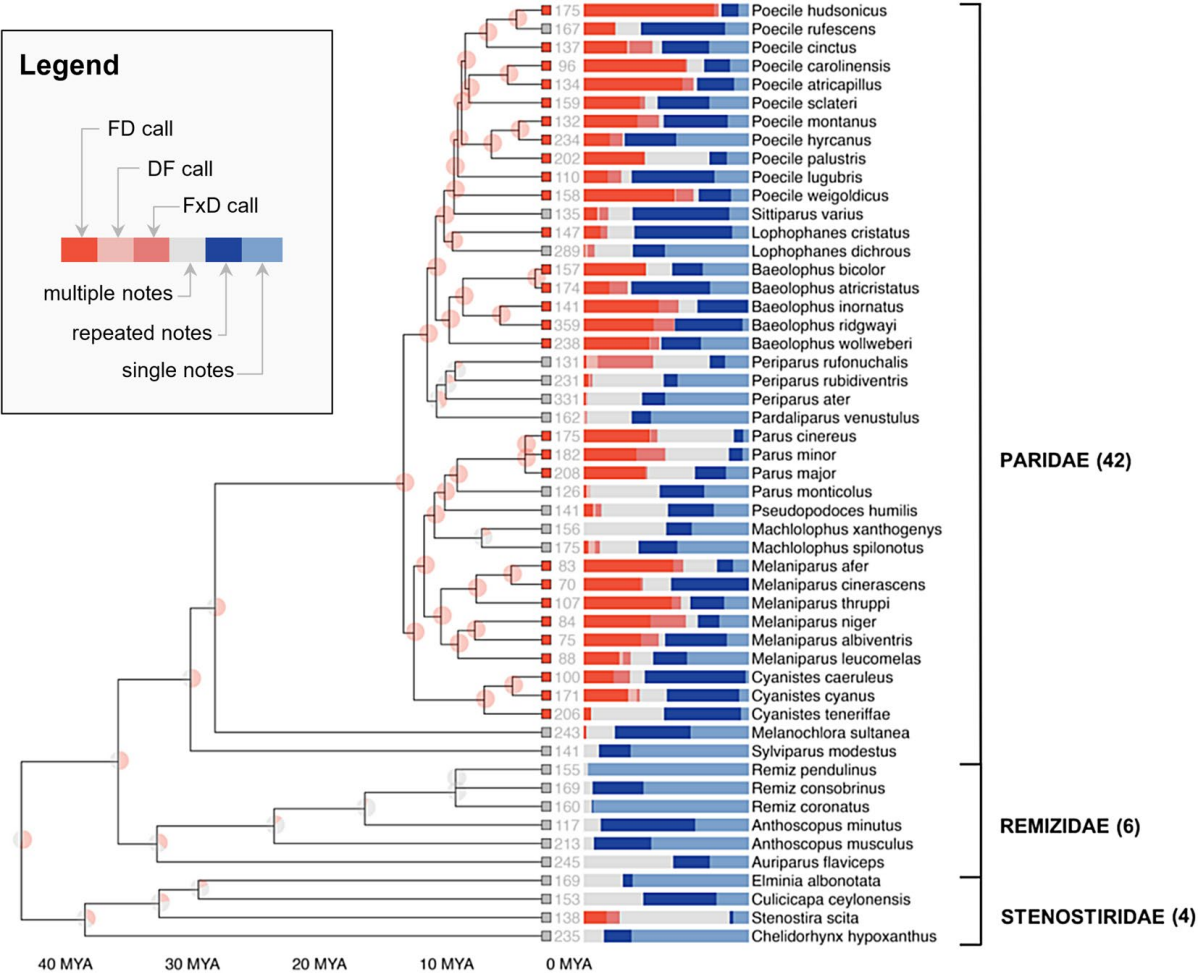
Phylogenetic tree of the bird species. The color of the squares at the tip of each branch represents whether the corresponding modern species passes the FD consistency test ( red) or not ( gray). The pie charts on the internal nodes represent the reconstructed probability that the ancestor species would pass the FD consistency test (=100% yes, =100% no). Numbers indicate the total number of calls in our dataset per species. The stacked bars represent the proportion of calls of the different types.

Call combinations were then categorized. A total of 1,621 calls (18.9%) corresponded to the so-called **FD calls**, defined as being made of a series of higher frequency and smaller bandwidth notes (F-notes), followed by a series of lower frequency and larger bandwidth notes (D-notes). Spectrograms of FD calls are given in Fig. 1 for visual reference. Also labeled were 52 **DF calls** (similar to the FD calls but in reverse order), 401 **FxD calls** (made of the two categories of notes but with more than one alternation between them) and 1,728 **other combination calls** (containing distinguishable notes, beyond just low and large vs. high and narrow notes). Figure 2 shows the proportion of each such combination per species in the phylogenetic tree.

A species was labeled as producing FD calls consistently if it satisfied the following criterion, which we call FD-consistency: we found at least one recording containing at least 5 calls, of which more than 50% were FD calls. This measure is conservative in that the existence of such a recording shows not only that the species produced some FD calls, but it also reveals consistency in its use both in form (keeping the order stable) and in context of use (suggesting that it is triggered by some event happening at the time of the given recording). 28 species passed the FD-consistency criterion, and all of them also turned out to be such that they produced at least 5 times as many FD calls as DF calls, also confirming the consistency in the use of order. By contrast, only one species passed the mirror DF consistency criterion, the Azure tit *Cyanistes cyanus*, but it also passed the FD-consistency test and in fact its recordings had more FD calls (*n* = 46) than DF calls (*n* = 9) or FxD calls (*n* = 3). Preference for FD calls over DF calls confirms the importance of order in the combination of F and D notes.

**Fig. 3:**
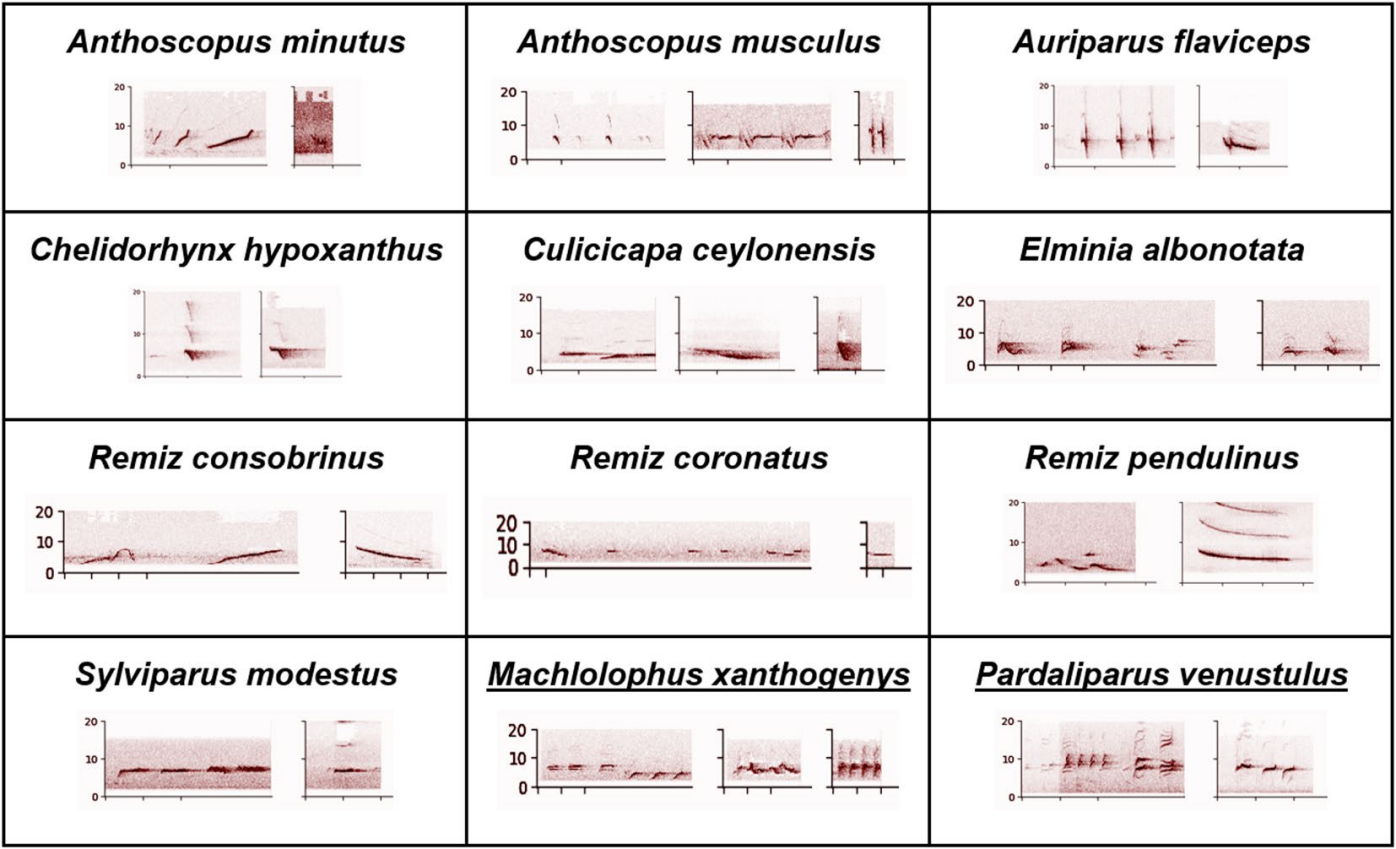
Examples of calls from the 12 species not producing FD calls consistently (the last two, underlined species are from the Paridae clade). The notes presented here in these calls are candidate F notes, at least in the sense that they seem to be much more similar to the F notes than to the D notes from the FD calls of species that do produce FD calls consistently. Spectrogram parameters: window size of 256 samples, overlap of 128 samples, FFT length of 256 points, power scaling factor of 0.6.

All 28 species passing the FD consistency criterion were from the Paridae clade. There were 12 species producing no FD calls at all, all outside of the Paridae clade. These species were also found not to produce DF calls nor FxD calls. We found some calls fitting our definition of FD calls in the call sequences of *Stenostrira scita* (one example given in Fig. 1), a species from the Stenostiridae family. However, this species did not pass the FD consistency criteria: the percentage of calls with an FD call organisation was always smaller than 50%. At the note level, calls made only of notes similar to D-notes were found in 41 out of 52 species, including all 28 species which passed the FD consistency criterion (but only in one species outside of the Paridae clade, *Remiz consobrinus*). Call sequences made solely of notes similar to F-notes were found in all species, whether they produce FD calls consistently or not. Since there is more variability in the characterization of the F-notes, we provide concrete examples of such calls in the methods section (Fig. 3), focusing on the species not producing FD calls consistently.

From there, we reconstructed the history of the FD call combination. A phylogenetic tree based on 13 published chronograms was obtained from DateLife^23–34^. It covered 51/52 species in our recordings (*Machlolophus aplonotus* could not be located in the phylogeny using the synthetic tree of Open Tree of Life^34^, accessed through the R package rotl^35^). We superimposed on that phylogenetic tree the binary trait recording presence/absence of FD calls in each species. Using the rerooting method of Yang and colleagues^36^, we fitted an equal rates (ER) model and an All Rates Different (ARD) Model. We compared the models using the AIC and BIC scores, two standard measures which aim to strike a balance between model complexity and fit to the data. The ARD model reached a better BIC score (54.2 vs. 59.2) and AIC score (50.3 vs. 57.3). The inferred transition rates under the ARD model showed strong asymmetry of the rates: the rate of appearance of FD call combinations was 15 times lower than the rate of disappearance. We thus report results from the ARD model (similar ancestral values are obtained with the ER model). We reconstructed the distribution of our binary trait at ancestral nodes. In particular, we conclude that there is a **99.8% probability that FD call combinations were used by the common ancestor of Paridae 11.2 million years ago.** Going further back in the tree, there is almost no information on the value of the trait for deeper nodes: the probability decreased to a 55.1% probability 26.1 million years ago (the age of the common ancestor between the Paridae and *Melanochlora Sultanea*), 49.7% 33.7 million years ago for the common ancestor of the Paridae and Remizidae, and 55.5% at the most recent common ancestor to all species in our sample, 41.3 million years ago.

To ensure that this result was robust, we ran simulations in which any recording may be ignored with a probability of 50% (as if these recordings had been misclassified for instance), or in which we arbitrarily assumed that an FD species could be a non-FD species with a 10% chance (which would typically require discarding many more recordings). For each of these tests we ran 1,000 simulations, and in 95% of each of these sets of simulations we obtained a probability of an FD call in the ancestor of Paridae above 95%. Furthermore, we ran an analysis in which all of the 6 species that passed the FD consistency test only based on a single recording were counted as not passing the FD test, and there again the probability of the FD calls existing in the ancestor of the Paridae was estimated to be above 99%.

To our knowledge, this is the oldest order- and content-dependent combination of signals that has been traced in the animal kingdom.

## Discussion

Using phylogenetic and comparative methods, we argue that FD combinations arose between 11 and 26 million years ago. Between 10 and 15 million years ago, the geographical historical reconstruction of the Paridae place the ancestors of this clade in the eastern Himalayas^37^. We can therefore reasonably estimate the emergence of the FD calls in the mountainous regions of East Asia. By comparison, whether human language existed as recently as half a million years ago is hotly debated [e.g.^38,39^]; regarding the presence of specific words or word combinations at that time, current research is unable to provide definitive answers.

Whenever a combination of calls is meaningful, there are two questions to ask: how is the meaning of this combination derived (in other words, what is its semantics)? and how is the combination produced in the first place (in other words, what is its syntax)?

On the **meaning** side, several hypotheses could be entertained about these combinations. First, they could be a mere acoustic coincidence (a kind of phonological syntax^40^), with FD being only acoustically related to F and D. To take a point of comparison: in English, the adjective *irate* is made of the sounds of the pronoun *I* and of the verb *rate*, but this is an accident: *irate* is neither morphologically nor syntactically made of *I + rate*, as the meaning has nothing to do with a rating. On this ‘acoustic coincidence’ view, the use of FD is essentially unrelated to the use of F and D. While this view cannot be excluded, a potential difficulty with it is that in several contemporary species, the number of F and D notes can be varied independently [e.g.^41^], which suggests that F and D have distinct roles in the sequence.

Second, ancestral combinations could be interpreted merely as the conjunction of two meanings (in the same way that, in English, *It is hot. It is humid*. involves the conjunction of the meanings of the independent sentences). On this view, the meaning of FD is in essence F and D, and it is an instance of ‘trivial compositionality’ (see^18^ for discussion). One question that could be debated pertains to the individual meaning of F and D notes. In the Japanese tit and the great tit, it has been hypothesized that F notes have an alerting function, while D notes have a recruitment function. These possible meanings are notably aligned with the motivation- structural rule of Morton: D notes, with their sharp onset, and with both low and high frequencies, are highly localizable^42^, while high frequency pure tones (like many F notes) are often identified as anti-predator flee calls. In other species, the core meaning of F and D notes has resulted in more complex outcomes. For example, in Carolina chickadees, one subcategory of F notes (called the C notes,^43^), were associated with flight behaviours, also in feeding contexts: the combination of F calls and D calls may not solely pertain to the conjunction of alarm and recruitment calls across the phylogenetic tree of Parids. Importantly, this question about the core meaning of the calls does not invalidate the hypothesis of trivial compositionality: the question of the meaning of the isolated units is different from the question of how these units are organized.

Third, these ancestral combinations could involve non-trivial compositionality, in the sense that the meaning of FD is derived from the meanings of F and of D but is not reducible to their mere conjunction, and is obtained only for a specific, recognizable organization (as is the case in English with *(it is) increasingly hot*, which does not mean that it is increasing(ly) and it is hot). While this seems to be the most sophisticated hypothesis, precisely this analysis has been proposed on the basis of multiple experiments in Japanese tits^15–17^. They have yet to be extended to other Parids for a complete reconstruction of this possibility along the phylogenetic tree. Complex combinatorial abilities have also been demonstrated in other Parid species^44,45^, although the changes in meaning appear to be related to the different proportions of notes, rather than their strict combination, which may indicate a different mechanism.

On the **syntactic** side, the robust asymmetry between the FD order and the mirror DF order needs to be explained. Here too, there are several hypotheses to be entertained. One line of analysis involves acoustic constraints. Some could be on the production side. For instance, there are anatomical and neuronal constraints on the two parts of the syrinx that may lead to high frequency sounds, usually produced by the right part of the syrinx, to be produced first^46^. Other constraints could be on the perception side. For instance, the Fs may precede the Ds because otherwise F notes may be perceptually masked by the broadband frequencies of D note^47^, or because higher frequency notes have more chance to trigger the attention of potential receivers^48^.

A second line of analysis involves the syntax proper: there could be an irreducible rule that specifies that FD is well-formed and D-F is ill-formed (for instance, in English, *former colleague* is well-formed but *colleague former* is ill-formed*).* Such an analysis would be in line with the view of Suzuki and colleagues about Japanese tits, since for them non-trivial compositionality and a non-trivial syntax are involved.

A third line of analysis involves the pragmatics (the optimal use of calls) rather than the syntax. For instance, it was proposed for several species that the calls that are more urgent should come before calls that are less urgent^1^, with the consequence that an alert call should come before a recruitment call. If the individual meanings of the F notes are confirmed to be more urgent than the individual meanings of the D notes, this pragmatic principle may be applicable.

In principle, detailed comparative work might help decide among these competing hypotheses, both on the semantic and on the syntactic side. It may also be very likely that several mechanisms are responsible for the emergence of such a combination. If arguments for non-trivial compositionality can be found for species located in sufficiently diverse parts of the phylogenetic tree, we might conclude that the ancestral FD sequences also involved non-trivial compositionality. This information is currently absent from the literature. Several examples may, in fact, suggest that other species, such as North American chickadees, perceive their FD calls differently than the system proposed by Suzuki et al. for the Japanese tit [e.g.^40^]. If this perspective is validated, a new question will emerge: When did non-trivial combinations arise in the ancestors of Japanese tits, and what were the underlying reasons? An even more ambitious question will be: Why, from a natural selection perspective, did non-trivial compositionality arise? (for possible answers, see^49^). The fact that other species outside the Paridae, such as *Stenostira scita*, can also produce similar types of combinations may also contribute to this inquiry, by exploring the ecological and evolutionary factors shared between these species. In conclusion, the demonstration that F and D notes were combined at least 11 million years ago is a stimulating first step for future work exploring the evolution of coding mechanisms in birds.

## Methods

All materials and code for analyses are available at:

https://osf.io/rjsfw/?view_only=947c5c6f94714168a77ebe6ea8ebd2d9.

### Selection of species and acoustic materials

We selected our recordings from the large-scale participative repository, xeno-canto.org. Launched in 2005, xeno-canto allows anyone to upload their bird recordings. It hosts more than 870,000 recordings as of June 2024. For each recording uploaded on the platform, the contributors can add details such as its geographical position, altitude, date, type of recording, or type of call/song. Contributors can also include comments to specify some information on the context surrounding the recording (type of forest, notable stimuli, etc.). While this information is extremely useful, it does not completely ensure a lack of bias in the recordings. For example, a large proportion of the recordings present on xeno-canto are recordings in which the human observer is near the caller, possibly influencing its calling behavior. In addition, some parts of the repertoire (e.g., low amplitude calls for short-distance communication) are likely to be underrepresented.

We developed a protocol to detect the FD combination in a large number of species with a low type I error (i.e. we strive to avoid concluding that FD calls are used by a species which, in fact, does not produce this combination). Given the limitation of the repository, type II errors (not detecting that a species produces FD calls) may occur. We organized our analyses with the same sampling effort for each species, allowing us to provide analyses that are not biased in this regard (i.e. we may not detect species that rarely produce FD calls, but this risk is the same throughout the phylogenetic tree). Note also that given that FD calls are often associated with mobbing behavior, such repositories may possess a positive bias to detect FD combinations (i.e. mobbing calls are loud and often associated with conspicuous behavior, hence more easily recorded). As a baseline, we also used the same method to detect DF calls, which turned out, as expected, to be much less frequent. Finally, we used a stringent criterion we called “FD-consistency” to label a species as reliably producing an FD call (at least 50% of FD calls in at least one recording with more than 5 calls, see main text). We also showed that the results are robust to even more stringent criteria (e.g., categorizing species as having FD calls if they also had 5 times more FD calls than DF calls, or imposing that the criterion on one recording was passed for at least 2 of the 10 recordings of a species). We take this to be conservative, because even a plain absence of a recording does not guarantee that a species does not produce FD calls (if more recordings were to be analyzed). Finally, we also labelled calls as FD in a conservative manner in the first place, in that some researchers may judge more call combinations as being of the FD type (for example, Hailman would only include the high versus low frequency differences to cluster the two types of notes, 17). As a result, conclusions about the presence of the FD combination in ancestor species are also conservative: if anything, we may underestimate their age.

There are 64 species in the Paridae^50^, 11 species in the Remizidae and 9 species in the Stenostiridae. We selected the species for which we could download 10 recordings that fulfilled the following three characteristics: (i) more than 10 notes in the recording, (ii) only one individual calling (or other individuals clearly in the background, with a lower sound to noise ratio), (iii) no strong noise in the background. We chose to use a sample size of 10 recordings per species as a trade-off between high chances of detection and the desire to include as many species as possible. To select our recordings, we discarded the files labeled as ‘songs’ and classified the xeno-canto files by quality (hence using the recordings with the best sound-to-noise ratio). We selected the first 10 recordings which fulfilled the three characteristics described above. We obtained 10 recordings for 52 species: 42 Paridae, 6 Remizidae and 4 Stenostiridae. For *Sylviparus modestus*, we only obtained 9 recordings, but we chose to keep this datapoint because of the importance of this species in the phylogeny of Paridae (at the edge of the clade). Overall, we obtained 519 recordings, summing up to 6 hours and 17 minutes.

### Creation of the datasets

Each recording was visualized with Avisoft SAS Lab by a first investigator (AS, an ornithologist with 6 years of experience), who first segmented the sequences into calls. We followed the definition of Collier and colleagues^51^ to segment the calls: combinations were considered as such when the notes were clearly separated from the preceding and following notes by a longer silence than those separating the notes within the combinations. This was done first by eye by AS, with the assistance of software to pre-segment the files (function *segment*, package *soundgen*). We then confirmed the stability of our segmenting system by extracting 10 intra- and 10 inter-call intervals from each species post-hoc: the intra call intervals were of 0.09 ± 0.10 s (mean ± SD), and inter call intervals were of 2.55 ± 2.39 s. When plotting the distribution of the inter-call and intra-call interval for each species, the intra- and inter-call intervals never overlaped, demonstrating the cohesiveness of our choices. For each of these calls, the first investigator (AS) then counted on the spectrogram the number of notes in the call (continuous traces on the spectrograms associated with the amplitude pattern), whether the call involved different note types and if so, whether this combination was of the FD type. FD calls were defined as a combination of two homogeneous series of notes, with the notes from the first series (F-notes) of higher frequency and smaller bandwidth than the notes in the second series (D-notes). The number of F or D notes in each series was not a factor in our analysis. Following this definition, [FDDDD] and [FFFFFFD] calls were FD calls, but [FFFDDDFF] and [DDDDF] calls were not. F and D notes could vary across renditions, even within a single call, as long as F notes were always higher and with a smaller bandwidth than the D notes. Previous work on a subset of Paridae has shown that detection by eye was efficient with the same level of accuracy as with a machine learning program trained on the spectrograms of the notes, and that frequency and bandwidth were the two parameters with stable differences between F and D notes^52^. Such a simple definition may overlook the occurrence of FD calls in certain species, as it does not account for possible variations in the acoustic properties of their F and D notes. We adhered strictly to this conservative definition; this may increase our risk of failing to identify FD calls in some species, thereby enhancing the robustness of any positive findings.

To confirm the analysis of the first investigator, three additional investigators, non-specialists in ornithology (< 1 year of experience), were trained to follow the same procedure on a subset of the recordings. We gave them the simple definition provided above and three examples from three species for which the FD call organization had been described. The three investigators had to analyze the calls of 10 different species (1,511 calls): three species for which the first investigator determined low proportions of FD calls, three species with high proportions of FD calls, and four species for which the proportions of FD calls were intermediate. The investigators were provided with the Latin names of the species; however, we are confident that it did not influence their labelling. The species names were unfamiliar to the investigators, and none of these species were present in the investigators’ home country. The investigators had to note whether each call was (i) a combination of notes, and (ii) specifically, whether it was an FD call. The average disagreement score across investigators (number of disagreements with the first investigator divided by the number of calls analyzed) was 5.66%. Disagreements were higher for the identification of combinations by the first coder (3.17%, 8.20%, and 9.86%) compared to calls identified as FD calls (3.71%, 2.97%, 6.02%). This is not surprising since no specific criterion to determine whether notes were from the same type or not was given, allowing different choices (e.g., a common source of disagreement was two notes with similar frequency features but dissimilar durations). The agreement scores did not vary much across the 10 species, ranging from 2.28 to 8.51%.

As a final post-processing step, some calls were found to have negative duration due to typos, or to overlap with one another. The first investigator corrected these data points manually. The investigator also added finer-grained information about calls being made of F and D notes, but possibly in a different order: DF calls, or FxD calls (F and D notes alternating more than once). We obtained a dataset for which the final characteristics are added in (Table 1).

**Table 1:** Quantitative information on the dataset used to analyze the presence of the FD combination in paridae, Remizidae and Stenostiridae.

Total number of recordings		519
Total duration of the recordings		6h17
Total duration of the calls in the recordings		1h17
Number of calls	**Total**	**8** **562**
	FD calls	1621
	DF calls	52
	FxD calls (other orders)	401
	Calls made of multiple notes (other)	1728
	Calls made of one repeated note	2210
	Calls made of one note	2550

### Examples of calls

Figure 3 shows examples of notes from the 12 species not producing FD calls. These notes resemble the F-notes produced in the FD calls.

## Data Availability

All materials and code for analyses are available at: https://osf.io/rjsfw/?view_only=947c5c6f94714168a77ebe6ea8ebd2d9.
